# The potential role of YAP in head and neck squamous cell carcinoma

**DOI:** 10.1038/s12276-020-00492-9

**Published:** 2020-08-28

**Authors:** Eunbie Shin, Joon Kim

**Affiliations:** Graduate School of Medical Science and Engineering, Korea Advanced Institute of Science and Technology (KAIST), Daejeon, Korea

**Keywords:** Head and neck cancer, Cell signalling

## Abstract

The transcriptional cofactor YAP and its inhibitory regulators, Hippo kinases and adapter proteins, constitute an evolutionarily conserved signaling pathway that controls organ size and cell fate. The activity of the Hippo-YAP pathway is determined by a variety of intracellular and intercellular cues, such as cell polarity, junctions, density, mechanical stress, energy status, and growth factor signaling. Recent studies have demonstrated that YAP can induce the expression of a set of genes that allow cancer cells to gain a survival advantage and aggressive behavior. Comprehensive genomic studies have revealed frequent focal amplifications of the *YAP* locus in human carcinomas, including head and neck squamous cell carcinoma (HNSCC). Moreover, *FAT1*, which encodes an upstream component of Hippo signaling, is one of the most commonly altered genes in HNSCC. In this review, we discuss the causes and functional consequences of YAP dysregulation in HNSCC. We also address interactions between YAP and other oncogenic drivers of HNSCC.

## Head and neck cancer: Protein dysfunction may give cancer the edge

Abnormal activity of a protein involved in cell proliferation may influence the progression of head and neck cancers. Head and neck squamous cell carcinoma (HNSCC) affects the skin, throat, mouth and nose tissues. Disruption to the Hippo-YAP signaling pathway, which plays a key role in cell proliferation and differentiation, is implicated in multiple cancers. Joon Kim and Eunbie Shin at the Korea Advanced Institute of Science and Technology, Daejeon, South Korea, reviewed recent research into the role of YAP in HNSCC. Abnormal YAP protein activity triggers the expression of genes that encourage cancer cell proliferation. Mice with over-expressed YAP showed tissue overgrowth and tumor formation. High YAP levels have been found at the invasive front of HNSCC tumors, suggesting a role in metastasis. Further research is needed to verify whether YAP is a potential therapeutic target.

## Introduction

**Fig. 1:**
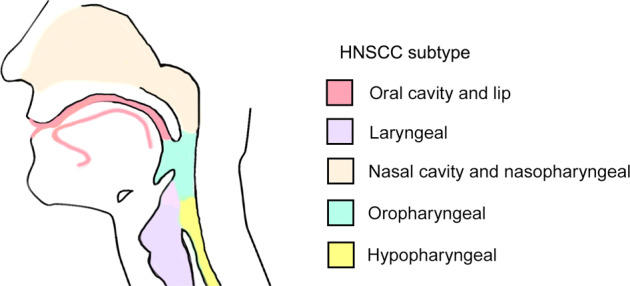
HNSCC subtypes according to anatomical location. Oral cavity cancer is found in the buccal mucosa, oral tongue, alveolar ridge, floor of the mouth, hard palate, and retromolar trigone. Laryngeal cancer is found in the supraglottis, vocal cords, glottis, and subglottis. Oropharyngeal cancer is found in the soft palate, base of the tongue, tonsillar pillars, and tonsillar fossa.

Head and neck cancer refers to a heterogeneous group of malignant neoplasms arising in the mucosal linings of the upper aerodigestive tract^1–3^ (i.e., the lips, oral cavity, tongue, salivary glands, larynx, nasal cavity, paranasal sinuses, and pharynx; Fig. 1). It was the seventh leading cancer by incidence worldwide in 2018^4^. The majority of head and neck cancers are squamous cell carcinomas. The oral mucosa and the surface of the tongue consist of areas of keratinized and nonkeratinized stratified squamous epithelium^5^. The pharynx and the vocal cord of the larynx are coated by nonkeratinized stratified squamous epithelium, and the rest of the larynx is lined by ciliated pseudostratified columnar epithelium. In all these epithelia, stem or progenitor cells that proliferate and produce differentiated cell types are present in the basal cell layer^6^. Postmitotic cells in suprabasal layers of a stratified squamous epithelium progressively flatten before being lost. Lineage tracing studies in mice have provided evidence that cancer cells originate from the basal cell layer^6,7^. Sequential accumulation of genetic alterations in basal cells is thought to generate preneoplastic lesions that progress into invasive carcinomas.

The primary risk factors for the development of HNSCC are tobacco use and heavy alcohol consumption^1^. Chewing betel leaves with areca nuts, which is common in Southeast Asia and the Indian subcontinent, is another known risk factor. Inherited diseases such as Fanconi anemia and Li-Fraumeni syndrome increase susceptibility to HNSCC^8,9^. In the Western world, infection with high-risk human papilloma viruses (HPVs) accounts for the increasing incidence of HNSCC that arises in the oropharynx^10,11^. Advances in surgery and radiation therapy have increased the cure rate of patients with early-stage HNSCC^1–3^. Aggressive approaches that combine chemotherapy, surgery, and/or radiation therapy are used to improve the outcome of patients with locally advanced HNSCC. For recurrent or metastatic HNSCC, the efficacy of these curative-intent treatments is limited, and the prognosis is very poor. HPV-positive oropharyngeal carcinoma is more susceptible to chemotherapy and radiation therapy and has a better prognosis than HPV-negative HNSCC. There are molecularly targeted therapy options approved for the treatment of HNSCC as a monotherapy or combination therapy. The monoclonal anti-EGFR antibody cetuximab shows modest long-term benefit in a small percentage of HNSCC patients^12^. The overall survival rate can be further improved by the immune checkpoint inhibitors pembrolizumab and nivolumab, which block PD-1 signaling, but only a fraction of patients with advanced HNSCC still exhibit significant therapeutic responses^13^.

The Hippo-YAP pathway plays an important role in the regulation of cell proliferation and differentiation during embryonic development and tissue regeneration^14,15^. A number of studies have indicated that the dysregulation of this pathway is deeply implicated in cancer pathogenesis across multiple cancer types^16–18^. Recently, comprehensive genomic characterization of HNSCC tumors revealed recurrent alterations in genes encoding Hippo-YAP pathway components^2,19^. In this review, we provide a brief overview of the role of YAP in cancer and discuss the potential contribution of Hippo-YAP pathway dysregulation to the development and progression of HNSCC.

## Somatic genetic alterations in HNSCC

Genomic data from over 500 HNSCC patients are available through The Cancer Genome Atlas (TCGA) database^20^. An analysis of the data revealed recurrent somatic mutations and copy number alterations in HNSCC tumors^2^. Missense or truncating mutations in the *TP53* tumor suppressor gene are the most frequent of all somatic genetic changes in HNSCC. Approximately half of HNSCC patients carry a deletion or truncating mutation in the *CDKN2A* gene, which encodes tumor suppressors p16/INK4A and p14/ARF, which regulate the cell cycle^21^. Mutations in or amplifications of *PI3-kinase catalytic subunit α* (*PIK3CA*) that can induce the overactivation of the AKT pathway are also frequently found in HNSCC patients^22^. In addition, TCGA Pan-Cancer Atlas analysis found amplifications of genes encoding EGFR and the cell cycle regulator Cyclin D1 in 11% and 24% of HNSCC patients, respectively. *NOTCH1* and *TP63*, which are involved in squamous cell differentiation during development and homeostasis, were also identified as significantly mutated genes^23,24^. Consistent with the fact that HPV E6 and E7 proteins inactivate p53 and the p16-Cyclin D-RB pathway, alterations in the *TP53* and *CDKN2A* genes are not frequent in HPV-positive HNSCC^25^. By contrast, as in cervical cancer, genetic alterations of PI3-kinase signaling constitute a strong driver for HNSCC, independent of HPV involvement.

**Fig. 2:**
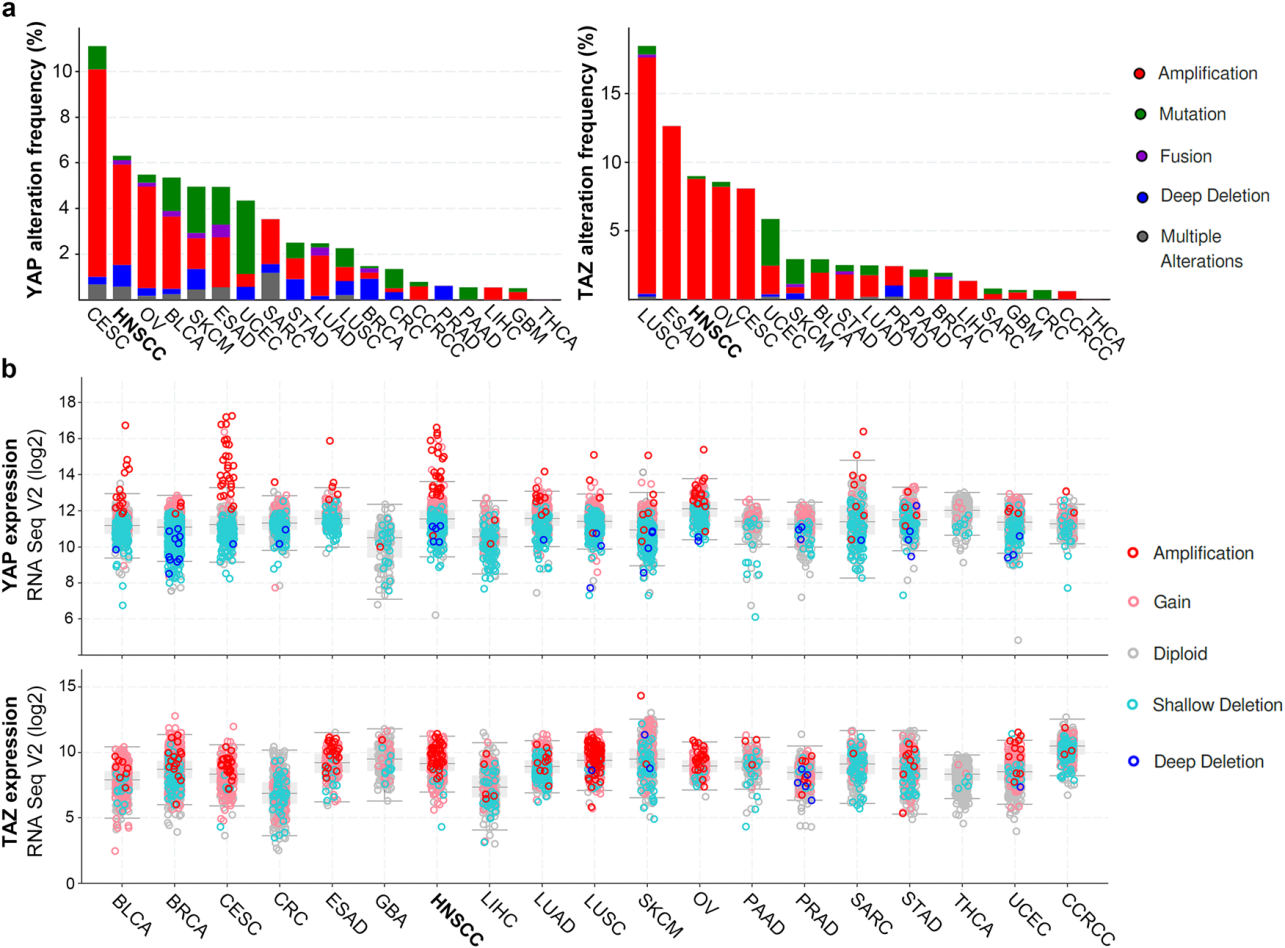
Genetic alteration in and expression of *YAP* and *TAZ* in human cancers. **a** The frequency of alterations in *YAP* and *TAZ* in different cancer types. **b** YAP/TAZ mRNA expression (RNA Seq V2) in various types of cancer. Each color represents different types of somatic alterations. TCGA PanCancer Atlas data were retrieved with cBioPortal. CESC cervical squamous cell carcinoma, HNSCC head and neck squamous cell carcinoma, OV ovarian serous cystadenocarcinoma, BLCA urothelial bladder cancer, SKCM cutaneous melanoma, ESAD esophageal adenocarcinoma, UCEC uterine corpus endometrial carcinoma, SARC sarcoma, STAD stomach adenocarcinoma, LUAD lung adenocarcinoma, LUSC lung squamous cell carcinoma, BRCA breast invasive carcinoma, CRC colorectal adenocarcinoma, CCRCC clear cell renal cell carcinoma, PRAD prostate adenocarcinoma, PAAD pancreatic ductal adenocarcinoma, LIHC liver hepatocellular carcinoma, GBM glioblastoma, THCA thyroid cancer.

Mutations in the *YAP* gene are not frequent, but recurrent YAP1-MAML2 and YAP1-NUTM1 fusions have been identified as activators of Hippo-YAP pathway signaling in skin cancers and several other cancer types^26,27^. In addition, recurrent focal amplification of 11q22, which contains the *YAP* gene, occurs in some types of carcinomas, including HNSCC (Fig. 2a). In HNSCC, *YAP* gene amplification predominates in HPV-negative tumors. However, mutual exclusivity between YAP and HPV infection is not clear in that *YAP* gene amplification is also frequent in cervical cancer caused by HPV infection in most cases. *YAP* gene transcription levels are well correlated with *YAP* gene amplification status in HNSCC and cervical cancer (Fig. 2b). TAZ (also known as WWTR1) is the only paralog of YAP. YAP and TAZ share significant homology and domain structures and are similarly regulated by Hippo signaling^28^. YAP and TAZ have overlapping transcriptional targets but are not completely redundant and have some distinct functions^29^. The amplification of the *TAZ* gene is frequent in HNSCC and some other types of cancers (Fig. 2a). *TAZ* gene amplification is not generally predicted as an oncogenic driver event because *TAZ* is mostly coamplified with a nearby potent oncogenic driver, the *PIK3CA* gene. The expression of *TAZ* is correlated with gene amplification, but *TAZ* overexpression is not as pronounced as the overexpression of the *YAP* gene (Fig. 2b). FAT1 is known as an upstream component of Hippo signaling, and functional loss of FAT1 can cause the activation of YAP and TAZ^30^. Somatic loss-of-function mutation or deletion in the *FAT1* gene is a recurrent event across human cancers, and HNSCC is one of the cancer types with the highest rate of alterations in this gene^31^. These cancer genomics data and recent functional studies suggest that YAP and TAZ play an important role in the pathogenesis of HNSCC.

## The core components of the Hippo-YAP pathway

**Fig. 3:**
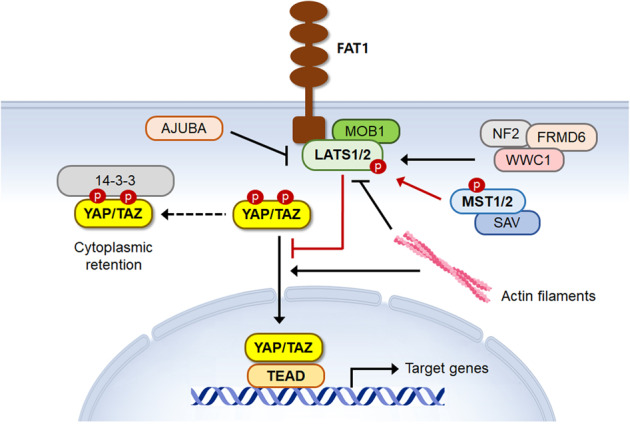
Schematic representation of core components of the Hippo-YAP pathway. YAP and TAZ are regulated by Hippo kinases and their adapters. FAT1 supports the assembly and activation of Hippo signaling components. LATS-mediated phosphorylation promotes cytoplasmic retention of YAP and TAZ.

Genes constituting the Hippo-YAP pathway were first discovered by genetic screens in *Drosophila* as organ size regulators^14,28,32^. The serine/threonine protein kinases Hippo and Warts and their adapter proteins Salvador and Mats are the core Hippo signaling components in *Drosophila*. The transcriptional coactivator Yorkie was identified as an effector that links Hippo signaling with the transcriptional control of organ size. Subsequent studies revealed orthologs of the *Drosophila* genes that constitute the mammalian Hippo-YAP pathway (Fig. 3). The serine/threonine protein kinases MST1 and MST2 (also called STK4 and STK3) are mammalian orthologs of *Drosophila* Hippo. MST1 and MST2 phosphorylate another core Hippo kinase, LATS1 and LATS2, the Warts orthologs. Phosphorylation by MST1/2 activates LATS1/2, which, in turn, phosphorylates multiple serine residues of YAP and TAZ, the Yorkie orthologs. LATS1/2-mediated phosphorylation causes cytoplasmic sequestration of YAP and TAZ by promoting the binding of the 14-3-3 proteins. Phosphorylation by LATS1/2 can also induce proteasomal degradation of YAP and TAZ. Target recognition and kinase activity of MST1/2 and LATS1/2 are assisted by SAV1 (Salvador ortholog) and MOB1A/B (Mats orthologs), respectively. When the core Hippo kinases are inactive, YAP and TAZ translocate into the nucleus. YAP and TAZ do not directly bind to DNA but bind to the TEA domain family member (TEAD) transcription factors and act as transcriptional coactivators.

There are four *TEAD* genes in the human genome. TEADs are broadly expressed and have been shown to play an important role in development, tissue homeostasis, and tumorigenesis^33^. YAP/TAZ are the major regulators of TEAD transcriptional activity. The binding of YAP/TAZ promotes the transcription of TEAD target genes. The vestigial-like (VGLL) protein family VGLL1-4 also interact with TEAD. YAP/TAZ and VGLL competitively bind TEAD, and VGLL binding inhibits YAP/TAZ-TEAD target gene expression^34–36^. In addition to YAP/TAZ and VGLL binding, the activity of TEAD is regulated by subcellular localization and posttranslational modifications^33,37^. Although TEADs are the central mediator, the transcriptional output of YAP and TAZ can be modulated by their interaction with multiple transcriptional regulators, such as AP-1, RUNX, SMAD, TBX5, BRD4, and the intracellular domain of ERBB4^14,38^.

## Regulation and function of the Hippo-YAP pathway

The core Hippo kinases and adapters integrate signaling from diverse upstream cues to control the activity of YAP and TAZ instead of relying on a dedicated ligand-receptor pair that acts as a molecular switch^14,28,32,39^. In addition, recent studies demonstrated that YAP and TAZ are influenced by Hippo-independent regulators that are activated by mechanical or biochemical stresses^40^. The architecture and tension of the actin cytoskeleton is a key determinant of Hippo-YAP signaling activity^41^. In epithelial cells, Hippo-YAP signaling is mainly regulated by several multimeric protein complexes associated with the apical plasma membrane or cell–cell junctions. In general, adhesion to a soft extracellular matrix, the formation of polarized epithelial sheets, or compaction at high cell densities activates the Hippo kinases, resulting in the inhibition of YAP and TAZ. Conversely, increased extracellular matrix stiffness and the loss of apical-basolateral polarity of epithelial cells, which are commonly observed in cancer, promote the activation of YAP and TAZ. Therefore, the Hippo-YAP pathway allows cells to sense extracellular matrix stiffness and cell polarity, adhesion, density, and shape.

NF2 (also called Merlin) is a membrane-cytoskeleton scaffolding protein^42^. Mutations in the *NF2* gene cause neurofibromatosis type 2, a disorder characterized by the growth of noncancerous tumors in the nervous system. NF2 and WWC1 (also called KIBRA) form an apical membrane-associated complex that recruits the core Hippo kinases to the apical plasma membrane and promotes MST1/2-mediated activating phosphorylation of LATS1/2 (Fig. 3). FRMD6 (also called EX1 or Willin) is another membrane-cytoskeleton scaffolding protein involved in the regulation of the Hippo-YAP pathway^43^. FRMD6 physically interacts with NF2-WWC1 and stimulates LATS1/2 activation^44^. NF2 localized at adherens junctions interacts with α-catenin to promote LATS1/2 activation^45^. The LIM domain-containing protein AJUBA can also recruit LATS1/2 to adherens junctions in response to cytoskeletal tension^46^. Unlike NF2, AJUBA inactivates the recruited LATS1/2 and consequently activates YAP and TAZ. FAT1 is a member of the cadherin superfamily, and the extracellular portion of FAT1 contains 34 cadherin repeats. A recent study demonstrated that FAT1 enables the assembly of a multimeric Hippo signaling complex, promoting the activation of the core Hippo kinases^30^. Loss-of-function mutations and the deletion of the *FAT1* genes may be associated with unrestrained activation of YAP in HNSCC.

Mechanical cues such as extracellular matrix stiffness and cell shape can affect the activity of YAP and TAZ through the contractile actin cytoskeleton^47^. Rho GTPases, key regulators of actin polymerization dynamics, and several other actin-interacting proteins have been shown to affect the activity of YAP and TAZ^41,48^. In addition, G-protein-coupled receptors that can control actin polymerization have been identified as regulators of YAP and TAZ. Rho GTPases and polymerized contractile actin bundles strongly inhibit LATS1/2 activity, but the molecular mechanism is not yet clear. The actin cytoskeleton can also affect YAP and TAZ by restricting the function of AMOT, which directly inhibits YAP and TAZ^49^.

The Hippo-YAP pathway plays an essential role in controlling cell proliferation and fate decisions in mammalian embryos^28,32,44,45^. YAP and TAZ are required for the maintenance of proliferative potential during development, but in adult tissues, the growth-promoting potential is usually suppressed in an inactive state by the action of the core Hippo kinases. YAP and TAZ are reactivated during tissue repair by the disruption of the normal tissue architecture and inflammation^15,32^. Ectopic activation of YAP can induce cell fate reprogramming of differentiated cells into linage-restricted stem cells in multiple cellular contexts^50^. The hyperactivation of YAP and TAZ in normal hepatocytes was shown to cause massive p53-dependent cell senescence or death^51^. However, the activity of YAP and TAZ in cancer cells can trigger aggressive behaviors such as unrestricted proliferation, dedifferentiation, metastasis, and therapy resistance^16,17^. Targets of YAP and TAZ include genes involved in cell cycle regulation, mitosis, survival, and ribosome biogenesis. YAP and TAZ commonly occupy a subset of distal enhancers with the highest transcriptional outputs^52^. The binding of enhancers to YAP and TAZ promotes the recruitment of RNA polymerase II to target gene promoters through chromatin looping and interaction with the Mediator complex and BRD4, a member of the BET protein family that promotes oncogene expression^53^. YAP and TAZ also promote the release of RNA polymerase II from the proximal promoter pausing to enhance transcriptional elongation^52^.

## The expression of YAP and TAZ in HNSCC

In the normal stratified squamous epithelium, including the oral epithelium, the levels of YAP and TAZ proteins are generally low except for in the basal cell layer^54–57^. Immunohistochemical analyses of clinical samples have shown that YAP/TAZ expression extends beyond the basal cell layer of the oral epithelium in regions of severe dysplasia^54,57^. Significantly higher levels of YAP have been detected in invasive oral squamous cell carcinoma (OSCC) samples than in precancerous lesions^54,56,57^. Elevated TAZ levels are also observed in tongue OSCC samples^55,58^. YAP and TAZ have been proposed as poor prognosis markers. A study showed a significant association between TAZ abundance and the pathological grade of tongue squamous cell carcinoma^55^. OSCC patients exhibiting a higher YAP immunolabeling score show significantly poor survival rates^57^. However, the correlation between YAP expression levels and tumor grade was shown to be insignificant in HNSCC samples^54,59^. Decreases in the intensity of YAP immunostaining are observed in some higher grade tumors. The sample sizes of these studies are modest; therefore, further analyses are needed to verify the results. An analysis of clinical samples revealed that YAP subcellular localization is not uniform among HNSCC tumors^57^. YAP appears to be predominantly localized to the cytoplasm in well-differentiated HNSCC tissues, whereas nuclear or diffuse nuclear/cytoplasmic YAP distribution is observed in poorly differentiated tumors^56,60^.

Interestingly, the invasive front of the tumor shows stronger YAP expression than the proximal region of the tumor tissue, regardless of the pathological grade^54^. A recent single-cell transcriptomic analysis revealed intratumoral heterogeneity of HNSCC tumors in their expression signatures related to phenotypic features such as epithelial differentiation, partial epithelial-to-mesenchymal transition (EMT), hypoxia, and the cell cycle^61^. A subset of malignant cells expressing a partial EMT program was shown to localize to the invasive front of tumors in proximity to cancer-associated fibroblasts^61^. Classic EMT transcription factors, including SNAIL, TWIST, and ZEB, were not detected in the invasive front of primary tumors. YAP overexpression has been shown to support EMT in some cellular contexts^62–64^. Thus, it is conceivable that hyperactivated YAP in invasive front cells contributes to the induction of the EMT program. An association between YAP expression and HNSCC nodal metastasis was reported, suggesting an involvement of YAP in metastasis^54^.

Elevated levels of YAP expression have been observed in various human cancers, including cancers where *YAP* gene amplification is not frequent^17^. The correlation between the gene copy number and the transcript level of *YAP* in HNSCC samples indicates that gene amplification contributes to the elevation of YAP (Fig. 2b). However, other mechanisms may also function to upregulate the transcription of the *YAP* gene. GA-binding protein alpha (GABA) binds to the *YAP* promoter and activates *YAP* transcription^65^. HMGB1 is a nuclear protein that organizes chromosomes and regulates transcription. HMGB1 was shown to bind GABA to promote the transcription of *YAP* in hepatocellular carcinoma^66^. Although the involvement of GABA and HMGB1 in *YAP* expression control in HNSCC has not been addressed, the overexpression of HMGB1 observed in HNSCC suggests a potential role of the HMGB1-GABA complex in promoting *YAP* expression.

**Fig. 4:**
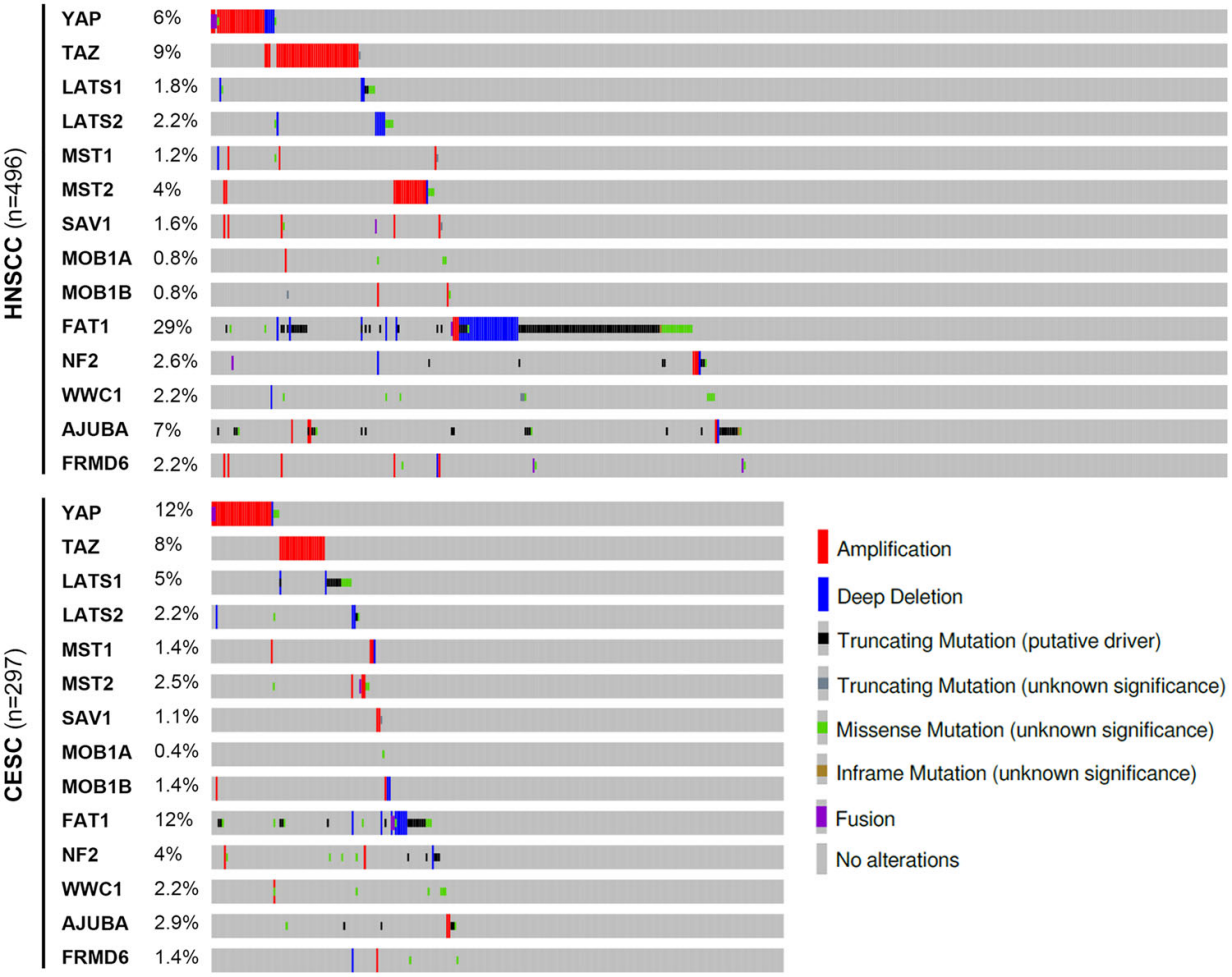
Genetic alteration in Hippo-YAP pathway genes. The proportion of somatic alterations in Hippo pathway genes in HNSCC and CESC samples. TCGA PanCancer Atlas data were retrieved with cBioPortal.

In general, nuclear translocation and protein stability are thought to be more important regulatory points for YAP and TAZ than transcriptional regulation^14,28^. Thus, alterations in the upstream regulators of YAP/TAZ proteins, especially Hippo signaling, are the prime candidates to mediate the dysregulation of YAP and TAZ in cancer. A recent study demonstrated that the deletion of *Mob1a/b* in mice rapidly induces the development of invasive OSCC through YAP hyperactivation^67^. However, with the notable exception of the FAT1 gene, loss-of-function mutations or the deletion of the genes encoding Hippo components are not frequently observed in human HNSCCs or cervical cancers (Fig. 4). It is not known why somatic alterations in *FAT1* are more frequent than alterations in the genes encoding the core Hippo kinases and their adapters in squamous cell carcinomas. The inactivation of *FAT1* has previously been linked to the activation of WNT signaling^68^. Currently, there is no conclusive experimental evidence that FAT1 genetic alterations contribute to HNSCC development through the overactivation of WNT signaling. In addition, WNT pathway genes, including the *APC* gene, are not frequently altered in HNSCC. A comprehensive meta-analysis of the Hippo pathway in multiple human cancers revealed high heterogeneity in the expression of Hippo pathway genes in squamous cell cancer types^31^, but a causal link between the downregulation of the expression of the core Hippo components and HNSCC development is unclear. The core Hippo kinases receive multiple upstream cues, and their activities are controlled by posttranslational modifications as well as subcellular localization; thus, the expression level of the genes alone may not accurately capture the pathway activity. Epithelial cell polarity cues are one of the major regulatory inputs to the Hippo-YAP pathway^44^. The disruption of epithelial polarity accompanying HNSCC formation may play a key role in derepressing YAP and TAZ.

## The role of YAP and TAZ in HNSCC

Studies using mouse models have demonstrated that the hyperactivation of YAP can induce tissue overgrowth and tumorigenesis. Transgenic overexpression of a mutated version of YAP (YAP-S127A) free from LATS-mediated cytoplasmic retention causes dramatic overgrowth of multiple tissues^69^. YAP-S127A overexpression in the mouse liver results in massive hepatomegaly, and long-term overexpression induces the formation of hepatocellular carcinomas^69^. In the intestine and skin, the expression of YAP-S127A expands the compartment of undifferentiated stem or progenitor cells^70,71^. Dysplastic changes in the tongue epithelium were also observed after short-term overexpression of YAP-S127A in the basal cell layer of stratified epithelia^71^. Tissue-specific knockout of the *YAP* and *TAZ* genes from the basal cell layer of the mouse skin suppresses the formation of papilloma and squamous cell carcinoma induced by chemical carcinogens^72^. Remarkably, the activation of YAP in the tongue through inducible deletion of the *Mob1a* and *Mob1b* genes causes the development of carcinoma in situ within 2 weeks and invasive squamous cell carcinoma within 4 weeks^67^. Tumorigenesis induced by *Mob1a/b* deletion is not affected by the loss of the *TAZ* gene, suggesting that YAP plays a major role in this genetic context. Cell lines established from the tongues of *Mob1a/b*-double-knockout mice exhibit enhanced self-renewal potential and chromosomal instability. These findings indicate that the dysregulation of endogenous YAP is sufficient to drive tumorigenesis in the oral epithelium^67^. YAP may confer cancer stem-cell-like properties observed in aggressive OSCC.

A number of reports using cell lines have also suggested that YAP and TAZ play a critical role in cancer pathogenesis in several cancer types, including HNSCC^73,74^. RNAi-mediated depletion of YAP and TAZ interferes with the proliferation, survival, colony-forming ability, and migration of aggressive OSCC cells^55,56,58,60^. Conversely, YAP overexpression promotes the proliferation and survival of OSCC cells. TAZ was shown to be involved in the EMT phenotype induced by TGF-β in OSCC cells^58,75^. It was also shown that tumor formation and the metastasis of OSCC cells xenografted into the mouse tongue require the function of YAP and TAZ^56^. Growing evidence indicates that YAP and TAZ can induce resistance to multiple anticancer treatment modalities, such as cytotoxic chemotherapy, molecular targeted therapy, and radiation therapy^76^. Suggested molecular mechanisms of YAP/TAZ-driven resistance include the upregulation of genes encoding growth factor signaling, anti-apoptosis genes, or DNA damage response genes. The induction of EMT is another proposed resistance mechanism promoted by YAP and TAZ activation. Cisplatin-resistant OSCC cell lines were shown to regain cisplatin sensitivity after YAP knockdown^77^. Another study demonstrated that TAZ is associated with cisplatin resistance in nasopharyngeal carcinoma cell lines^75^. An analysis of the intrinsic cetuximab sensitivity of HNSCC cell lines found that the amplification of the YAP locus is associated with cetuximab resistance^78^.

Downstream targets of YAP and TAZ are determined by their interactions with multiple transcriptional and epigenetic regulators whose expression and activity are dynamically modulated by oncogenic signaling. YAP/TAZ-regulated transcriptional signatures specific to each stage of HNSCC development and progression in vivo are not well known. The transcriptional signature regulated by YAP and TAZ in an OSCC cell line was identified by a study using siRNA-based YAP/TAZ knockdown and microarray analysis^56^. The study compared the SCC2 transcriptional signature in the OSCC cell line with that in TCGA OSCC patient data. Genes affected by YAP/TAZ knockdown were shown to be significantly correlated with gene expression changes occurring in human OSCCs, suggesting that a subset of genes dysregulated in OSCC patients is associated with YAP/TAZ activity. Another study also analyzed TCGA HNSCC data to test the significance of *YAP* amplification and overexpression^79^. A subgroup of HNSCC patients with *YAP* gene amplification and *YAP* overexpression showed worse prognosis across different HNSCC cohorts.

## Interaction of YAP and other oncogenic drivers of HNSCC

**Fig. 5:**
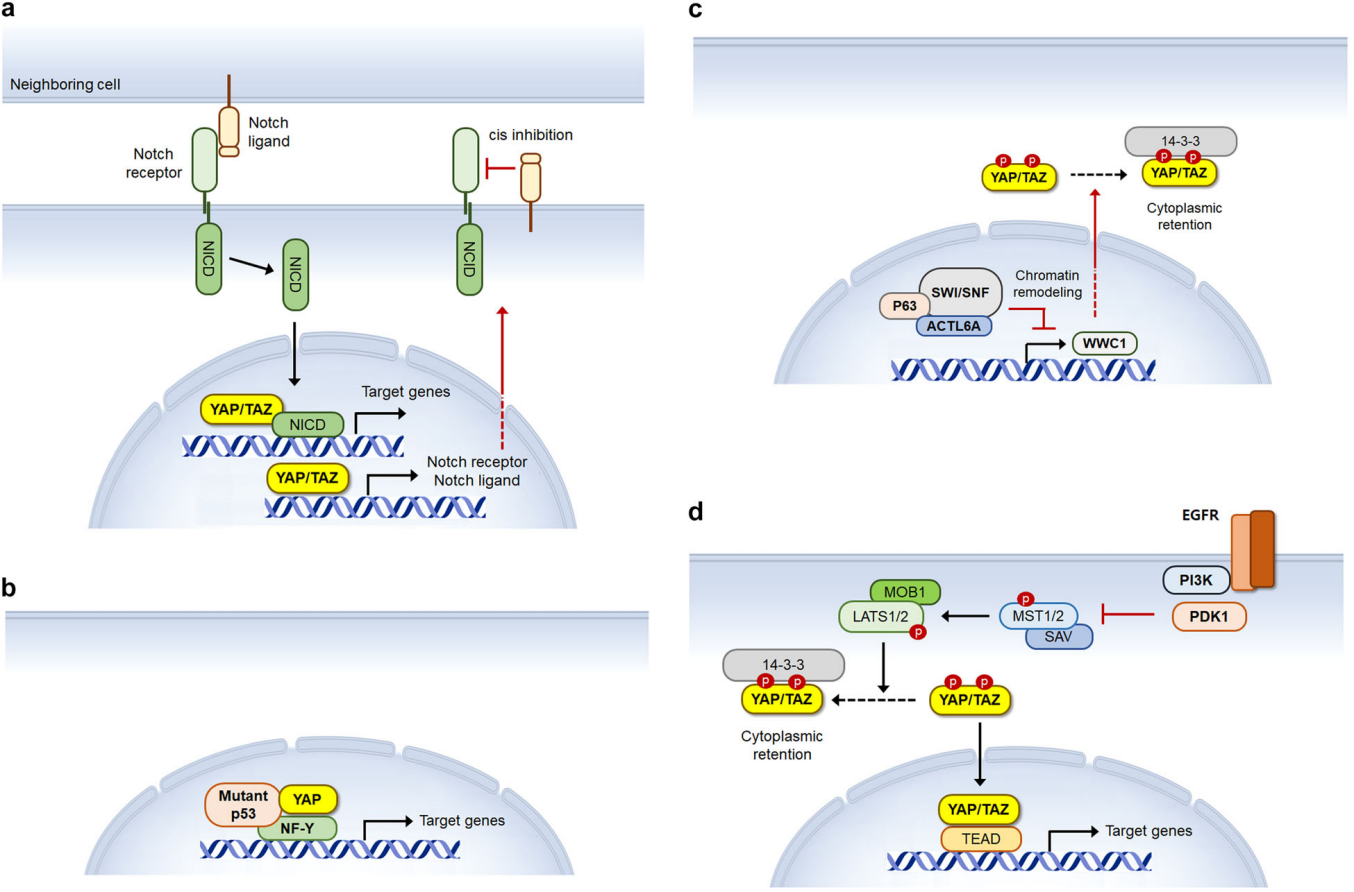
Schematic representation of interactions between YAP and other oncogenic drivers. **a** Activated YAP and TAZ regulate the expression not only of the common target genes along with NICD but also of Notch receptors and ligands. **b** The YAP/mutant-p53/NF-Y complex increases the expression of cell-cycle-related genes. **c** p63/ACTL6A-dependent chromatin remodeling promotes YAP/TAZ activation by suppressing *WWC1* expression. **d** Activated PI3K induces the dissociation of PDK1 from the core Hippo kinase complex, leading to the inactivation of Hippo signaling.

The binding of the transmembrane ligands Delta-like or Jagged to the Notch receptors of the neighboring cell induces the release of the Notch intracellular domain (NICD), which functions as a transcriptional regulator^24^. This juxtracrine signaling promotes keratinocyte differentiation in the suprabasal layer of the stratified epithelium^80^. In addition, Delta-like and Jagged proteins can induce cell-autonomous cis inhibition of Notch signaling in stem or progenitor cells in the basal cell layer to maintain their undifferentiated state. Most mutations in the *NOTCH1* gene in TCGA HNSCC cohorts are considered inactivating, indicating a tumor suppressor role of NOTCH1^19^. However, frequent activating *NOTCH1* mutations have also been detected by analyses of Asian HNSCC patients^81^. Not only diminished but also excessive NOTCH1 activity may promote tumorigenesis by disturbing the transition of keratinocytes from the basal to the suprabasal layers. YAP/TAZ-mediated transcriptional regulation appears to crosstalk with the Notch signaling pathway^82^ (Fig. 5a). Nuclear YAP/TAZ and NICD have been shown to interact to induce the expression of common target genes in vascular smooth muscle cells^83^. Moreover, in epidermal progenitor cells, YAP/TAZ activation by attachment to the basal lamina can drive the expression of Delta ligands, which induces cis inhibition of Notch signaling to suppress the differentiation of progenitor cells^84^. Further investigation of YAP/TAZ-Notch signaling interactions in HNSCC will contribute to a better understanding of the function of YAP/TAZ in the malignant transformation of squamous epithelial cells.

p53 plays a central role in tumor suppression by inducing cell death in response to cellular stresses^85^. A missense mutation in the *TP53* gene is a widespread molecular event in HNSCC^19^. Most of the mutations are inactivating, but some p53 mutants are thought to gain novel oncogenic function^86^. YAP was shown to directly interact with mutant p53 proteins to regulate the activity of the transcription factor NF-Y^87^. The YAP/mutant p53/NF-Y complex can promote tumor progression by enhancing the expression of cell-cycle-related genes, such as cyclins and CDK1 (Fig. 5b). The p53 family transcription factor p63 plays a key role in epithelial stratification during embryonic development and is also required for the maintenance of the basal cell layer in mature stratified epithelia^23^. Gene amplification and the overexpression of *TP63* are frequently observed in HNSCC. Unlike YAP and TAZ, which are upregulated in the invasive front of the tumor, most cancer cells in HNSCC tumors express high levels of p63 protein^54,59^. An isoform of p63, lacking the N-terminal transactivation domain (ΔNp63), is known as an oncogene that regulates transcriptional programs to sustain malignant cell proliferation and survival^88^. A recent study reported that p63 interacts with coamplified ACTL6A, a subunit of the chromatin-remodeling complex SWI/SNF. Chromatin remodeling by the p63/ACTL6A complex was shown to suppress the *WWC1* promoter^89^ (Fig. 5c). The downregulation of *WWC1* expression results in the activation of YAP and promotes tumorigenesis.

Epidermal growth factor receptors activated by its ligands EGF, TGF-α, or amphiregulin trigger the PI3-kinase signaling that promotes cell growth and survival. Genetic alterations in *EGFR* are relatively less frequent in HNSCC, but amplification of or mutation in *PIK3CA* is one of the most common oncogenic drivers in HNSCC^19^. Although the interaction between PI3-kinase signaling and YAP/TAZ in HNSCC has not been clearly addressed, activated PI3-kinase was shown to promote YAP activity by inactivating Hippo signaling in other epithelial cell lines (Fig. 5d). In normal resting cells, PDK1 interacts with and activates the core Hippo kinase complex^90^. Upon activation by ligand-bound EGFR, PI3-kinase recruits PDK1 to the plasma membrane, disrupting its interaction with the Hippo kinase complex. As a result, YAP is released from cytoplasmic retention. On the other hand, YAP/TAZ can activate EGFR signaling by inducing the expression of amphiregulin, which leads to the activation of PI3-kinase signaling^91^. Although further functional studies are needed, this positive feedback is likely to contribute to the activation of YAP/TAZ in HNSCC.

## Perspectives

Various lines of evidence have provided support for the significance of YAP and TAZ in cancer development and progression. Judging from the frequency of somatic genetic alterations in *YAP/TAZ* and *FAT1*, the dependence on YAP/TAZ hyperactivation is likely to be greater in carcinomas originating from stratified squamous epithelia than other cancer types. Further investigation is needed to understand the exact role of YAP and TAZ in the complex interplay among cancer genetics, epigenetics, and cell lineage. In this review, we highlight the cell-autonomous effects of the dysregulation of YAP/TAZ in malignant HNSCC cells. Recent studies not discussed here indicate that the YAP/TAZ-regulated transcriptional program is deeply involved in the establishment of a protumorigenic microenvironment^16^. YAP/TAZ activation in malignant cells can affect the behavior of other cells in the tumor microenvironment through the secretion of extracellular matrix components and cytokines. YAP-mediated recruitment of myeloid-derived suppressor cells and tumor-associated macrophages has been shown to lessen anticancer immunity^92^. Moreover, YAP and TAZ are activated in cancer-associated fibroblasts and regulatory T cells in the tumor stroma and promote their protumorigenic function^93,94^. Understanding the roles of YAP/TAZ in coordinating tumor-stromal interactions may offer new insight into HNSCC.

Advances in surgery and radiation therapy have improved the prognosis of early-stage HNSCC. However, patients with recurrent or metastatic HNSCC continue to show low long-term survival rates. Current chemotherapy and molecularly targeted therapy options are effective only in a small subset of patients. HNSCC is expected to be a promising target for upcoming anticancer drugs based on YAP/TAZ inhibition that can be used in combination with existing treatments. YAP and TAZ have been shown to induce resistance to the inhibition of the EGFR or the downstream RAS-MAP kinase pathway^76^. Therefore, combination therapy with an EGFR inhibitor and a YAP/TAZ inhibitor in HNSCC treatment is expected to have a synergistic effect. In addition, considering the emerging role of YAP/TAZ in immune suppression^94,95^, YAP/TAZ inhibitors may be promising candidates for combination therapy with anti-PD-1 therapy against recurrent and metastatic HNSCC.

Various research efforts are currently underway to develop YAP/TAZ inhibitors^96^. Because YAP and TAZ remain inactive in most healthy adult tissues, specific YAP/TAZ inhibitors may show a relatively low risk of side effects. Reagents that directly inhibit the interaction between YAP/TAZ and their transcriptional partner TEADs have been suggested to be effective in preclinical cancer models. For example, a peptide mimicking VGLL4 function blocked the YAP-TEAD interaction and suppressed the growth of gastric cancer in a mouse model^97^. Another strategy under investigation is to target the upstream regulators of YAP and TAZ. In addition, consistent with the physical interaction between YAP/TAZ and BRD4, the vulnerability of YAP/TAZ activity to BRD4 inhibitors has been demonstrated^53^. Together with an expanded understanding of the Hippo-YAP pathway in human cancer, the development of specific YAP/TAZ inhibitors with high potency can provide a new breakthrough in targeted therapeutic approaches to HNSCC.
